# Exploring the Toxicological Relationship Between Diisononyl Cyclohexane-1,2-dicarboxylate and Atherosclerosis Through Network Toxicology, Machine Learning, and Multi-Dimensional Bioinformatics

**DOI:** 10.3390/ijms27114668

**Published:** 2026-05-22

**Authors:** Jingbo Cao, Ziyao Yang, Qi Zhang, Siwei Zou, Huning Zhang, Anning Yang, Yue Sun

**Affiliations:** 1 General Hospital of Ningxia Medical University, School of Public Health, Ningxia Medical University, Yinchuan 750004, China; cao.jingbo@outlook.com (J.C.); yilin.2004@139.com (Z.Y.); 202304144040@nxmu.edu.cn (Q.Z.); 230240510143@nxmu.edu.cn (H.Z.); 2 The Second School of Clinical Medicine, Ningxia Medical University, Yinchuan 750004, China; 3 NHC Key Laboratory of Metabolic Cardiovascular Diseases Research, Ningxia Medical University, Yinchuan 750004, China; 24132z210112@nxmu.edu.cn; 4 Key Laboratory of Environmental Factors and Chronic Disease Control, Ningxia Medical University, Yinchuan 750004, China

**Keywords:** atherosclerosis, diisononyl cyclohexane-1,2-dicarboxylate, molecular docking, machine learning algorithm, molecular dynamics simulation

## Abstract

This study integrates multidimensional computational approaches—network toxicology, machine learning, molecular docking, and molecular dynamics simulation—to systematically elucidate the toxic mechanism by which the environmental pollutant diisononyl cyclohexane-1,2-dicarboxylate (DINCH) contributes to atherosclerosis. By jointly mining multiple databases, we obtained 246 targets common to DINCH and atherosclerosis. LASSO regression and support vector machine–recursive feature elimination (SVM-RFE) then identified 8 significantly upregulated core targets (CSF1R, CD36, CCL3, CCR2, ADAM8, TLR1, CTSS, and MMP1). Functional enrichment analysis showed that these core targets were significantly associated with key signaling pathways, including lipid and atherosclerosis, the PPAR signaling pathway, the PI3K–Akt signaling pathway, and the AGE–RAGE signaling pathway in diabetic complications. Differential gene analysis confirmed that these genes were significantly upregulated in diseased tissues, and receiver operating characteristic (ROC) analysis demonstrated excellent diagnostic performance (AUC = 0.87–0.96). Immune cell infiltration analysis further revealed a strong association between the core targets and immune cell populations, notably macrophages and T cells. Molecular docking and molecular dynamics simulations showed that DINCH had high affinity for the core targets, and its binding to CCR2 was the most stable (binding free energy = −7.6 kcal/mol). The final AOP framework systematically presented the cascade by which DINCH may contribute to atherosclerosis through metabolic disruption and immune activation. This study provides new mechanistic insights into the development of DINCH-induced atherosclerosis and offers a theoretical basis for health risk assessment of environmental pollutants.

## 1. Introduction

Atherosclerosis is a vascular disease largely driven by lipid metabolism disorders, chronic inflammation, and immune-response dysregulation, and its onset and progression are closely linked to various environmental risk factors [1]. Recent studies have shown that environmental pollutants can exacerbate atherosclerosis through multiple molecular pathways. For example, fine particulate matter (PM2.5) promotes monocyte infiltration by inducing endothelial dysfunction [2]; persistent organic pollutants, such as polychlorinated biphenyls (PCBs), disrupt lipid homeostasis via the aryl hydrocarbon receptor (AhR) signaling pathway [3]; and heavy metals such as cadmium (Cd) accelerate plaque formation through oxidative stress and vascular calcification mechanisms [4]. These exogenous chemicals often activate oxidative stress, endoplasmic reticulum stress, and epigenetic regulatory mechanisms [5], and they act synergistically with traditional risk factors to drive disease progression. Although the cardiovascular toxicity of the pollutants described above is relatively well characterized, research remains limited on the roles and mechanisms of many newer alternative pollutants, notably the recently widely used plasticizer diisononyl cyclohexane-1,2-dicarboxylate (DINCH) in atherosclerosis.

Diisononyl cyclohexane-1,2-dicarboxylate (DINCH; trade name Hexamoll^®^ DINCH^®^) is an important substitute for traditional phthalate plasticizers such as DEHP and DINP and is widely used in food packaging, medical devices, children’s toys, and textiles because of its relatively safer toxicological profile [6]. Since its initial launch in the European market in 2002, global annual production of DINCH rose rapidly from 25,000 tons to 200,000 tons by 2014, and the detection rate of its metabolites in human urine samples has increased significantly [7]. Although DINCH was initially considered a safe alternative because of low acute toxicity, accumulating in vivo and in vitro evidence indicates that long-term or low-dose exposure may still cause adverse health effects. Experiments showed that DINCH and its primary metabolite MINCH induced oxidative stress in macrophages and enhanced inflammatory responses at low concentrations. These compounds promoted the release of proinflammatory cytokines such as TNF-α and IL-1β by activating the NF-κB signaling pathway and may disrupt normal immune function [8]. In addition, Eljezi confirmed that both primary and secondary metabolites of DINCH exhibit significant cytotoxicity to L929 mouse cells [9]. However, the potential causal link and molecular mechanism between DINCH exposure and atherosclerosis have not been systematically investigated. The premise of this study is that DINCH may trigger oxidative stress and inflammatory responses, which are core pathological events in atherosclerosis. The theoretical rationale is that DINCH is structurally similar to phthalates, which are known to disrupt vascular lipid homeostasis and immune function; therefore, DINCH may contribute to atherogenesis through similar biological processes.

Methods such as network toxicology, machine learning, molecular docking, and molecular dynamics simulation are widely used to study toxic mechanisms [10]. Network toxicology integrates the compound–target–toxicity interaction network and reveals pollutants’ complex pathogenic mechanisms through systems biology and big-data analysis [11]. These computational strategies have been successfully applied to explore the toxicity of environmental pollutants and can efficiently identify key targets and molecular interactions related to cardiovascular diseases. Therefore, these approaches are suitable for revealing the potential association and mechanism of DINCH with atherosclerosis [12,13]. This study aims to explore the potential association between DINCH and atherosclerosis, identify core targets and key pathways, and provide a theoretical basis for environmental health risk assessment.

## 2. Results

### 2.1. ProTox and ADMETlab—Chemical Toxicity Prediction

We systematically predicted the toxicity profile of diisononyl cyclohexane-1,2-dicarboxylate (DINCH) using the computational toxicology platforms ProTox-3.0 and ADMETlab 2.0. The results indicated potential risks across multiple toxicity endpoints: organ toxicity mainly presented as drug-induced liver injury (DILI) (predicted probability: 0.707) and nephrotoxicity (predicted probability: 0.54), and the compound also showed potential carcinogenicity (probability: 0.77) and high skin sensitization (probability: 0.965) (Tables S2 and S3). In addition, DINCH exhibited high lipophilicity (predicted Log P = 7.09), indicating likely bioaccumulation and potential long-term exposure risks (Table S1). Given these toxicity characteristics, this study further applied a network toxicology approach to identify the molecular events by which DINCH modulates lipid metabolism, impairs endothelial cell function, and activates inflammatory pathways, thereby contributing to the pathogenesis of atherosclerosis. These are in silico predictive results that require further experimental validation and do not necessarily reflect real carcinogenic risk.

### 2.2. Network Toxicological Analysis of Potential Targets of DINCH-Induced Atherosclerosis

**Figure 1:**
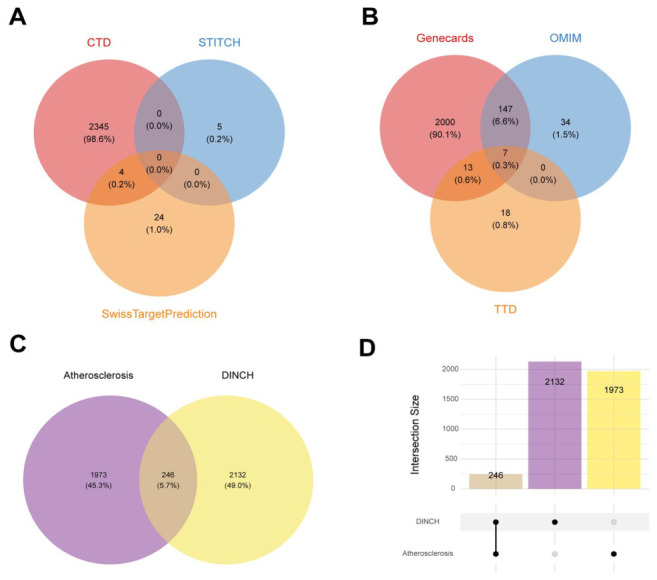
Venn diagram showing the action targets of DINCH and genes associated with atherosclerosis. The 246 overlapping targets in the diagram represent candidate targets for the potential atherogenic effects of DINCH (**A**–**D**).

Network toxicology is a systematic research method that can comprehensively predict the toxic mechanisms and molecular targets of environmental pollutants. In this study, we applied a network toxicology approach to explore the potential pathogenic association between DINCH and atherosclerosis. We integrated targets from the CTD, Swiss Target Prediction, and STITCH databases and, using a union method to merge entries and remove duplicates, screened a total of 2378 DINCH-related targets. Using the same procedure, we compiled 2219 unique atherosclerosis-related targets from the GeneCards, TTD, and OMIM databases. An intersection analysis of the two target sets was performed with a Venn diagram (Figure 1A–D), which identified 246 common targets. These targets may serve as potential mediators of DINCH-induced atherosclerosis.

### 2.3. Functional Enrichment Analysis of DINCH-Induced Atherosclerosis Targets

**Figure 2:**
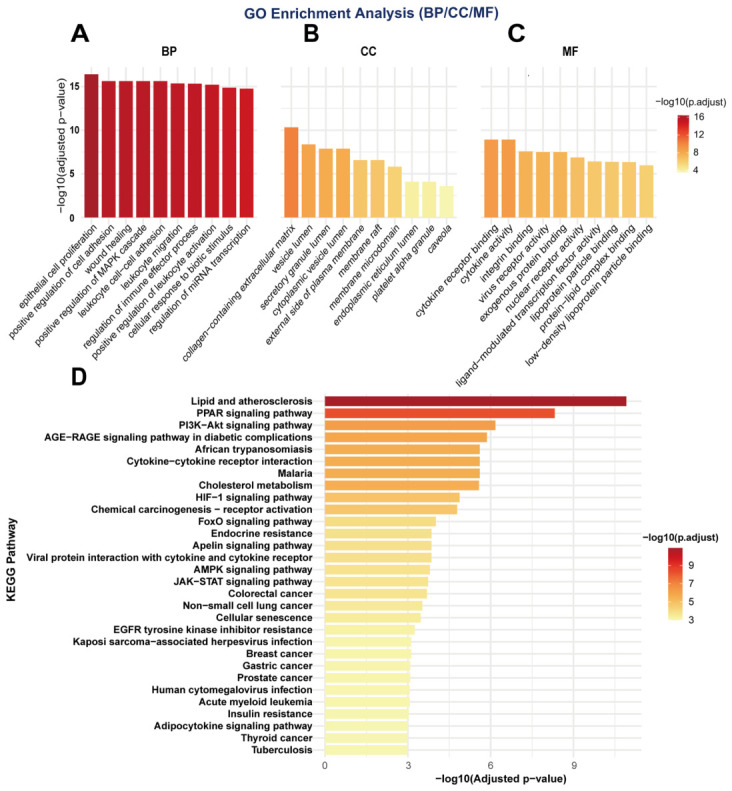
(**A**–**C**) Top 10 significantly enriched biological processes (BP), cellular components (CC), and molecular functions (MF), respectively. The color gradient of bars represents the -log10(adjusted *p*-value), with darker colors indicating higher statistical significance. (**D**) Top 30 significantly enriched KEGG pathways. The color gradient and bar length both correspond to the −log10(adjusted *p*-value), highlighting pathways with the strongest statistical enrichment. Enrichment analyses were performed using the clusterProfiler R package, with a threshold of adjusted *p*-value < 0.05. Numbers above the bars in panels (**A**–**C**) represent the −log10(adjusted *p*-value) for each enriched term.

Enrichment analysis of the 246 targets shared by DINCH and atherosclerosis preliminarily revealed potential pathogenic biological mechanisms. In the biological process (BP) category, target genes were significantly enriched in “leukocyte migration,” “leukocyte cell–cell adhesion,” and “positive regulation of leukocyte activation” (Figure 2A). These enrichments indicate the genes may jointly mediate directional recruitment, adhesion, and functional activation of immune cells, processes closely tied to the initiation of inflammation and immune responses. In the cellular component (CC) category, significant enrichment in “external side of plasma membrane,” “membrane raft,” and “secretory granule lumen” indicates the gene products may localize to the cell surface and secretion-related structures and thus participate in signal recognition and molecular secretion (Figure 2B). Molecular function (MF) analysis showed significant enrichment in “cytokine receptor binding” and “integrin binding,” which further supports roles for these targets in cell communication and inflammatory responses (Figure 2C).

Kyoto Encyclopedia of Genes and Genomes (KEGG) pathway analysis further revealed (Figure 2D) that these genes were significantly enriched in the “Lipid and atherosclerosis” core pathway. Enrichment was also observed in upstream regulatory pathways, such as the PPAR signaling pathway and the PI3K-Akt signaling pathway, and in signal transduction pathways, such as cytokine–cytokine receptor interaction. These results suggest that DINCH exposure may be associated with lipid metabolism disorders, activation of inflammatory responses, and insulin resistance, providing preliminary biological evidence for the hypothesis that DINCH may promote atherosclerosis development via these pathways.

### 2.4. Key Target Recognition Based on Machine Learning

**Figure 3:**
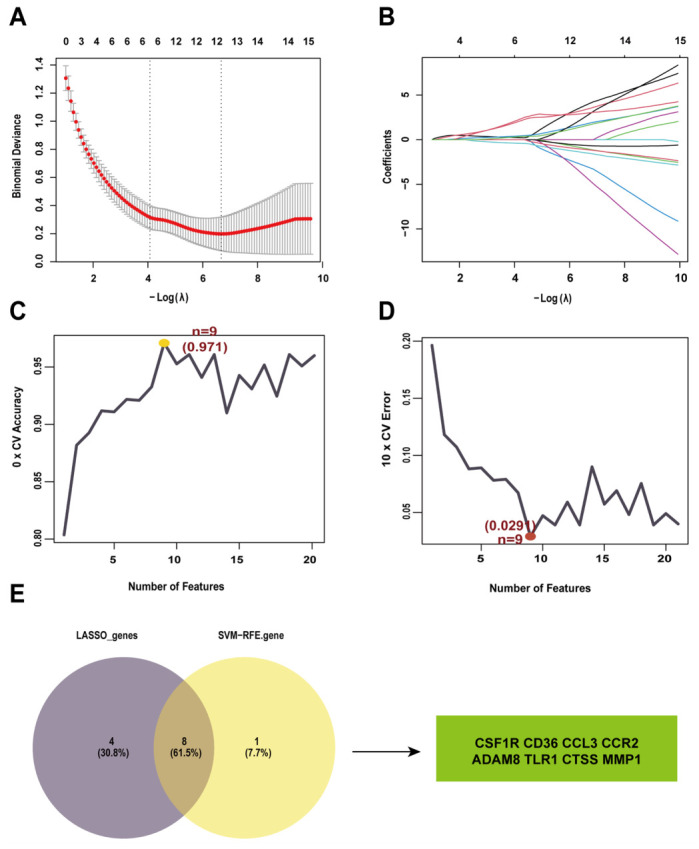
Screening of core target genes for diisononyl cyclohexane-1,2-dicarboxylate (DINCH)-induced atherosclerosis using machine learning algorithms. (**A**,**B**) LASSO logistic regression analysis: (**A**) Binomial deviance plot, where the red line represents the mean deviance and gray bars indicate standard error. The optimal λ value (lambda min) was selected to identify 12 candidate genes; (**B**) Coefficient profiles of LASSO regression, showing the shrinkage of gene coefficients with increasing −log(λ). (**C**,**D**) SVM-RFE analysis: (**C**) 10 × CV Accuracy curve, with the yellow dot marking the optimal feature number (*n* = 9, accuracy = 0.971); (**D**) 10 × CV Error curve, with the red dot marking the minimum error (n = 9, error = 0.029). (**E**) Venn diagram showing the overlap of genes identified by LASSO and SVM-RFE, with 8 overlapping genes defined as core targets for DINCH-induced atherosclerosis. All analyses were performed with 10-fold nested cross-validation to ensure model robustness.

To identify core genes among the 246 potential atherosclerosis targets related to DINCH, we combined least absolute shrinkage and selection operator (LASSO) regression with a support vector machine (SVM) algorithm. First, using the set of 246 targets, we applied LASSO logistic regression with nested cross-validation to optimize the regularization parameters and preliminarily selected 12 candidate core targets (Figure 3A,B). Next, we applied support vector machine–recursive feature elimination (SVM-RFE) to the same target set, which reduced the candidate list to 9 targets (Figure 3C,D). Finally, we intersected the gene sets from the two methods and visualized the overlap with a Venn diagram, yielding 8 high-confidence candidate core genes: CSF1R, CD36, CCL3, CCR2, ADAM8, TLR1, CTSS, and MMP1 (Figure 3E).

**Figure 4:**
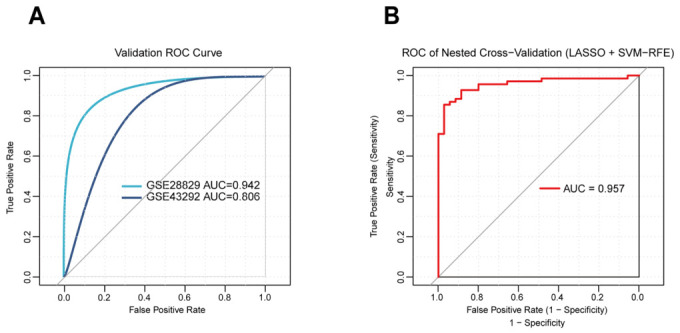
Receiver operating characteristic (ROC) curve analysis of the DINCH-associated atherosclerosis gene signature. (**A**) External validation ROC curves in two independent cohorts (GSE28829 and GSE43292). (**B**) Nested cross-validation (10 × 10) ROC curve in the training cohort (GSE100927). AUC values are provided in the legend for each curve; the diagonal line indicates an AUC of 0.5 (random classification).

We performed 10 × 10 nested cross-validation in the training cohort (GSE100927) to assess model stability, which yielded an area under the receiver operating characteristic curve (AUC) of 0.957 (Figure 4B). For external validation, we evaluated the gene signature in two independent atherosclerosis cohorts (GSE43292 and GSE28829) that were not used for feature selection or model training. The signature achieved an AUC of 0.942 in GSE28829 and 0.806 in GSE43292 (Figure 4A), suggesting potential diagnostic performance and generalizability across independent patient groups.

### 2.5. Expression Validation and Diagnostic Efficacy Test of 8 Key Targets for Atherosclerosis

**Figure 5:**
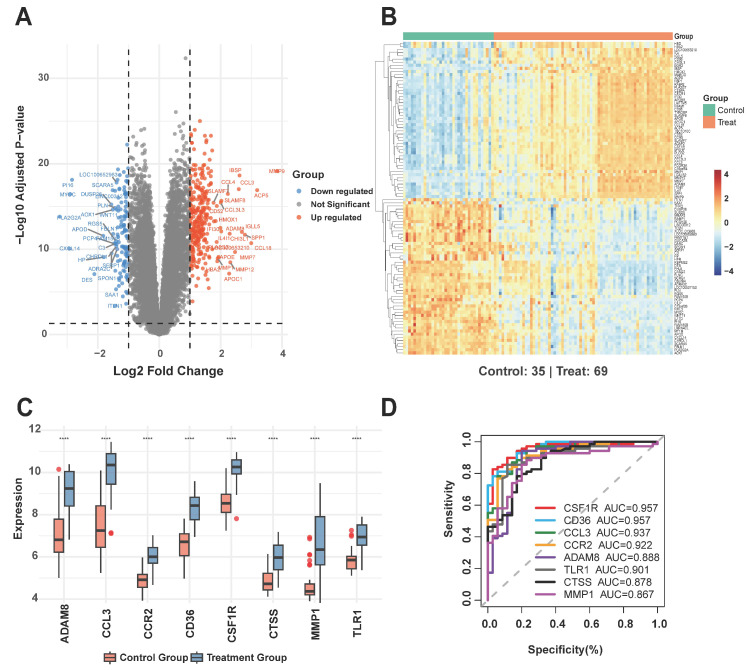
Expression validation and diagnostic efficacy analysis of core target genes in atherosclerosis. (**A**) Volcano plot of differentially expressed genes (DEGs) in the GSE100927 dataset (35 normal arterial vs. 69 atherosclerotic plaque samples). DEGs were defined as |log2FC| > 1 and adjusted *p* < 0.05 (dashed lines). Red: upregulated, blue: downregulated, gray: not significant. (**B**) Heatmap of top DEGs, with hierarchical clustering of samples and genes, color-coded by z-score normalized expression. (**C**) Box plots of normalized expression levels of 8 core target genes in control and atherosclerotic groups. Red dots represent outlier samples; **** indicates *p* < 0.0001 (Student’s *t*-test). (**D**) ROC curves evaluating the diagnostic efficacy of 8 core genes, with AUC values shown in the legend. The diagonal line represents a random classifier (AUC = 0.5).

The RNA-seq dataset (GSE100927) of patients with atherosclerosis was downloaded from the GEO database to validate the expression levels of a set of core genes. Compared with normal arterial tissues, CSF1R, CD36, CCL3, CCR2, ADAM8, TLR1, CTSS, and MMP1 were all significantly upregulated in atherosclerotic plaques (Figure 5C). To further assess the association between these genes and disease state, receiver operating characteristic (ROC) curves were plotted, and the area under the curve (AUC) values were calculated. The AUC values were 0.957 for CSF1R and CD36, 0.937 for CCL3, 0.922 for CCR2, 0.901 for TLR1, 0.888 for ADAM8, 0.878 for CTSS, and 0.867 for MMP1 (Figure 5D). All genes had AUC values greater than 0.85, which strongly indicated their high association with atherosclerotic disease. These findings underscore the research value of these eight core genes in the pathogenesis of atherosclerosis and provide a foundation for subsequent exploration of their molecular mechanisms and validation of their potential as diagnostic biomarkers in an independent clinical cohort.

### 2.6. Correlation Analysis Between Core Genes of Atherosclerosis and Immune Cells

**Figure 6:**
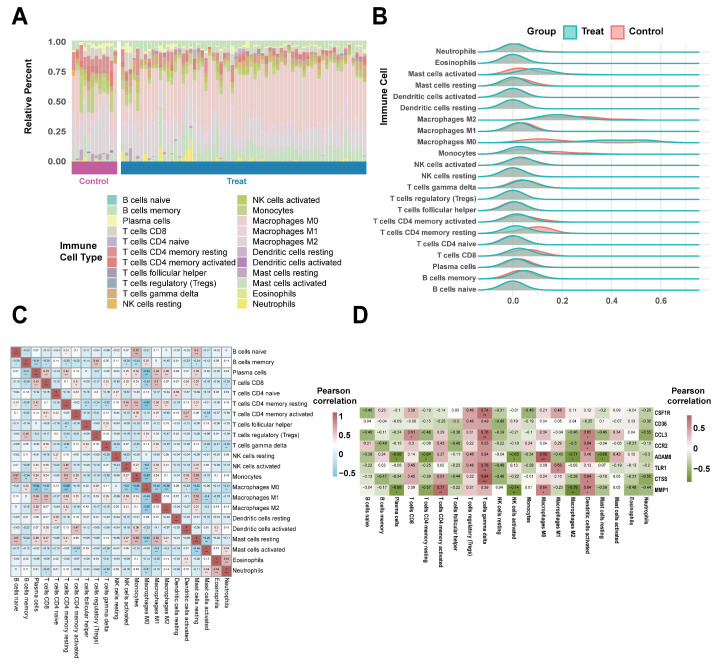
Immune infiltration analysis of atherosclerotic plaques and normal arterial tissues in the GSE100927 dataset (35 control vs. 69 atherosclerotic samples). (**A**) Stacked bar plot showing the relative proportion of 22 immune cell types, quantified by single-sample gene set enrichment analysis (ssGSEA). (**B**) Ridge plot comparing the abundance distribution of each immune cell type between the control (red) and atherosclerotic (teal) groups. (**C**) Pearson correlation heatmap of immune cell types in atherosclerotic plaques, with red indicating positive correlation and blue indicating negative correlation. (**D**) Pearson correlation heatmap showing the correlation between 8 core target genes and immune cell infiltration in atherosclerotic plaques. All correlation analyses were performed with *p* < 0.05 as the significance threshold. Significance levels are indicated as: * *p* < 0.05, ** *p* < 0.01, *** *p* < 0.001.

We used single-sample gene set enrichment analysis (ssGSEA) to quantify infiltration levels of 22 immune cell types in normal tissues and atherosclerotic plaque samples and systematically evaluated their correlations with the expression of eight core genes (CSF1R, CD36, CCL3, CCR2, ADAM8, TLR1, CTSS, and MMP1). The analyses showed altered infiltration of monocytes, M0 macrophages, M1 macrophages, mast cells, resting mast cells, and several T cell subsets (CD4+ resting memory T cells, CD4+ activated memory T cells, CD8+ T cells, and γδ T cells) (all adjusted *p* < 0.05, Figure 6A,B). A correlation heatmap further indicated distinct association patterns among immune cells: for example, activated CD4+ memory T cells correlated positively with activated dendritic cells, and activated B cells correlated positively with both resting mast cells and monocytes; in contrast, regulatory T cells (Tregs) correlated negatively with multiple proinflammatory subsets (activated CD4+ memory T cells, follicular helper T cells (Tfh), γδ T cells, and M0 and M1 macrophages), consistent with the well-known immunosuppressive role of Tregs (Figure 6C).

Further analysis of the relationship between core genes and immune infiltration showed that expression levels of CSF1R, CD36, CCL3, CCR2, ADAM8, TLR1, CTSS, and MMP1 were significantly positively correlated with infiltration scores of regulatory T cells and γδ T cells and negatively correlated with resting CD4+ memory T cells (Figure 6D). These results suggested a strong association between these core genes and infiltration of specific immune cells in the vascular immune microenvironment, providing a basis for subsequent functional experiments to investigate their potential mechanisms in atherosclerosis.

Notably, the ssGSEA algorithm was originally established and optimized for tumor transcriptomic data. When applied to non-neoplastic vascular tissues, the results should be interpreted with appropriate caution due to differences in tissue composition and immune contexture between tumors and atherosclerotic lesions.

### 2.7. Molecular Docking Analysis of DINCH with Atherosclerosis Targets

**Figure 7:**
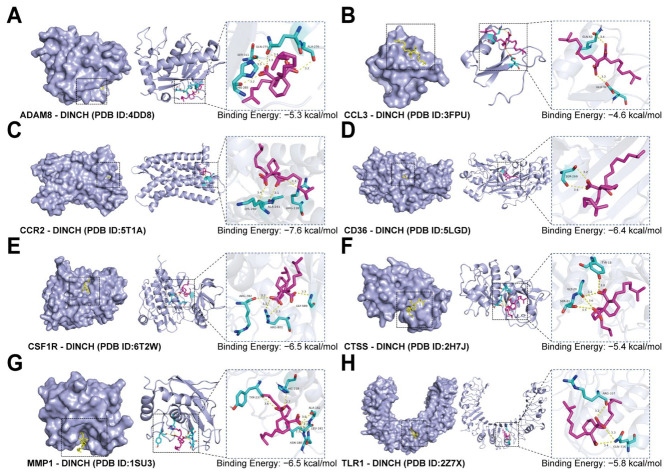
Molecular docking validation of the interaction between DINCH and core genes. (**A**) ADAM8-DINCH complex (PDB ID: 4DD8). Binding energy: −5.3 kcal/mol. (**B**) CCL3-DINCH complex (PDB ID: 3FPU). Binding energy: −4.6 kcal/mol. (**C**) CCR2-DINCH complex (PDB ID: 5T1A). Binding energy: −7.6 kcal/mol. (**D**) CD36-DINCH complex (PDB ID: 5LGD). Binding energy: −6.4 kcal/mol. (**E**) CSF1R-DINCH complex (PDB ID: 6T2W). Binding energy: −6.5 kcal/mol. (**F**) CTSS-DINCH complex (PDB ID: 2H7J). Binding energy: −5.4 kcal/mol. (**G**) MMP1-DINCH complex (PDB ID: 1SU3). Binding energy: −6.5 kcal/mol. (**H**) TLR1-DINCH complex (PDB ID: 2Z7X). Binding energy: −5.8 kcal/mol.

We used molecular docking to investigate interactions between DINCH and eight core target proteins (CSF1R, CD36, CCL3, CCR2, ADAM8, TLR1, CTSS, and MMP1) (Figure 7). Information on the receptor proteins is provided in Table S4. Docking produced binding free energies of −6.5, −6.4, −4.6, −7.6, −5.3, −5.8, −5.4, and −6.5 kcal/mol for these proteins, respectively (Figure S1). Binding free energies below 0 kcal/mol indicate thermodynamically spontaneous binding, while values below −5 kcal/mol often suggest favorable binding [14]. It should be noted that such thresholds are scoring-function-dependent and cannot be simply generalized. By these standards, DINCH bound most targets (CSF1R, CD36, CCR2, ADAM8, TLR1, CTSS, and MMP1) with energies below −5 kcal/mol, suggesting substantial binding, whereas CCL3 exhibited a higher energy (−4.6 kcal/mol) that did not reach the −5 kcal/mol threshold, indicating weaker binding. Notably, DINCH showed its lowest binding free energy with CCR2 (−7.6 kcal/mol), suggesting a moderate but favorable potential interaction.

### 2.8. Molecular Dynamics Simulation and Binding Free Energy Estimation

To address molecular docking’s inability to capture protein flexibility and environmental effects (temperature, pressure, solvent), we performed molecular dynamics (MD) simulations to evaluate the interaction stability of the CCR2–DINCH complex. From the MD trajectories, we analyzed root-mean-square deviation (RMSD), root-mean-square fluctuation (RMSF), radius of gyration (Rg), solvent-accessible surface area (SASA), and the number of hydrogen bonds between protein and ligand. We also examined the relative free-energy distribution, compared complex structures at 0, 25, 50, 75, and 100 ns, calculated the average binding free energy using the MM/GBSA method, and performed per-residue energy decomposition to identify key residues. CCR2 was selected for molecular dynamics simulation because it is a core chemokine receptor in the 8-gene signature, plays an essential role in monocyte recruitment and atherosclerotic vascular inflammation, and exhibits the most stable and strongest binding affinity in molecular docking. A blank control ligand was also included in the molecular dynamics simulation process to verify the specificity of binding between DINCH and target proteins.

**Figure 8:**
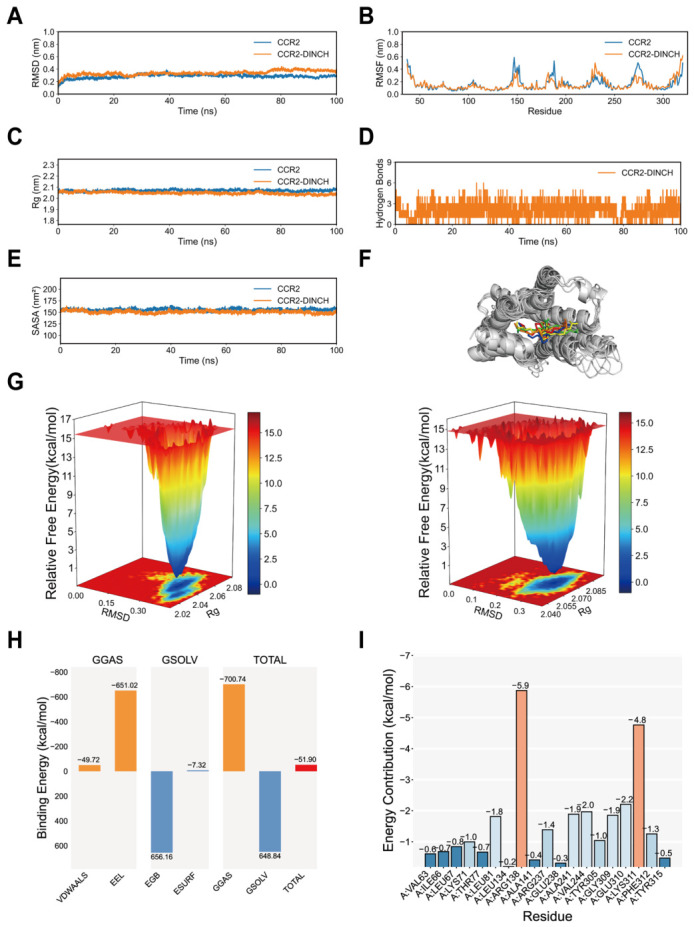
Molecular dynamics (MD) simulation analysis of the stability and binding affinity of the diisononylcyclohexane-1,2-dicarboxylate (DINCH)–CC chemokine receptor type 2 (CCR2) complex. All simulations were performed for 100 ns using GROMACS with the Amber force field. (**A**) Root-Mean-Square Deviation (RMSD) curves of the CCR2–DINCH complex and the apo CCR2, showing system equilibrium after 10 ns. (**B**) Root-Mean-Square Fluctuation (RMSF) curves of CCR2 residues in the complex and the apo form, reflecting regional flexibility. (**C**) Radius of Gyration (Rg) curves of the complex and the apo protein, indicating the compactness of the protein structure. (**D**) Fluctuation curve of the number of hydrogen bonds between CCR2 and DINCH, with the count mainly between 2 and 4 (maximum 6). (**E**) Solvent-Accessible Surface Area (SASA) curves of the complex and the apo CCR2, showing no significant change upon ligand binding. (**F**) Superimposed structures of the CCR2–DINCH complex at 0, 25, 50, 75, and 100 ns (colored red, green, blue, yellow, and orange, respectively), demonstrating stable binding of DINCH in the orthosteric pocket. (**G**) Free energy landscapes of the apo CCR2 (**right**) and the CCR2–DINCH complex (**left**), showing a single lowest-energy cluster for apo CCR2 and two closely distributed energy minima for the complex. (**H**) Average binding free energy of the CCR2–DINCH complex calculated by the MM/GBSA method. VDWAALS, EEL, EGB, ESURF, GGAS, GSOLV, and TOTAL represent van der Waals, electrostatic, polar solvation, non-polar solvation, gas-phase, solvation, and total binding free energy, respectively. (**I**) Per-residue energy contributions of key amino acids in CCR2 involved in DINCH binding.

The simulation results showed that the complex’s root-mean-square deviation (RMSD) fluctuated steadily between 0.3 and 0.45 nm and remained below 1 nm throughout the simulation, nearly overlapping with the RMSD curve of the apo CCR2, suggesting good overall structural stability (Figure 8A). Root-mean-square fluctuation (RMSF) analysis revealed that the CCR2 protein’s RMSF values stayed within 1 nm without notable peaks, indicating that DINCH binding did not produce significant perturbations in the CCR2 structure (Figure 8B). The radius of gyration (Rg) remained stable at approximately 2.05 nm with a smooth trajectory, almost overlapping with that of the apo protein, reflecting that the complex retained a compact, nonexpanded conformation (Figure 8C). Hydrogen bond analysis showed that the number of hydrogen bonds between CCR2 and DINCH fluctuated mainly between 2 and 4 (with a maximum of 6), suggesting a continuous and stable hydrogen-bond network (Figure 8D). The solvent-accessible surface area (SASA) remained near 160 nm^2^ with minimal variation and closely matched the apo CCR2, further supporting the folding stability of the protein (Figure 8E). By comparing complex conformations at five time points during the molecular dynamics simulation, we found that DINCH occupied the same binding site on CCR2 at 0, 25, 50, 75, and 100 ns without significant change, indicating favorable binding stability (Figure 8F). Free energy landscape (FEL) analysis showed that the apo CCR2 formed a single lowest-energy cluster, whereas the CCR2–DINCH complex exhibited two closely distributed energy minima, indicating that the complex adopted a highly stable, confined conformational ensemble (Figure 8G).

Furthermore, the average binding free energy of the CCR2–DINCH complex calculated by MM/GBSA was −51.90 kcal·mol^−1^, suggesting stable binding affinity (Figure 8H). The total binding free energy (TOTAL) was decomposed into van der Waals (VDWAALS), electrostatic (EEL), polar solvation (EGB), and non-polar solvation (ESURF) contributions. Per-residue energy decomposition revealed that residues ARG-138 (−5.9 kcal·mol^−1^), LYS-311 (−4.8 kcal·mol^−1^), GLU-310 (−2.2 kcal·mol^−1^), and VAL-244 (−2.0 kcal·mol^−1^) played dominant roles in the interaction (Figure 8I). These key residues are consistent with the binding pocket identified by molecular docking, suggesting that DINCH remained anchored at the same site throughout the simulation with high stability. Together, these metrics provide preliminary atomic-level evidence for the formation of a stable, well-behaved complex between CCR2 and DINCH.

### 2.9. Construction of Adverse Outcome Pathways

**Figure 9:**
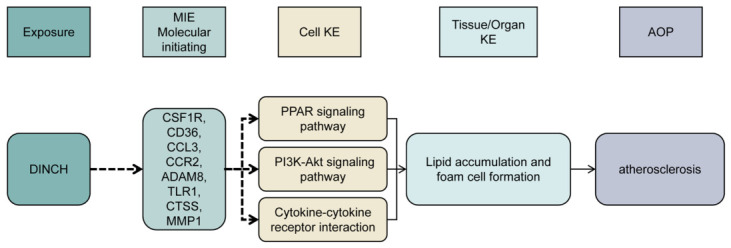
Hypothesis of adverse outcome pathway (AOP) for DINCH-induced atherosclerosis. Solid lines = literature-supported key event relationships; dashed lines = predicted/in silico relationships. This framework is conceptual, preliminary, and inferential, requiring further experimental validation.

Based on the method proposed by Daniel Villeneuve [15], we developed a new adverse outcome pathway (AOP) framework. The framework indicates that DINCH may disrupt biological processes, including lipid metabolism and atherosclerosis, the PPAR signaling pathway, cytokine–cytokine receptor interactions, and the chemokine signaling pathway, by altering the expression and function of key targets such as CSF1R, CD36, CCL3, CCR2, ADAM8, TLR1, CTSS, and MMP1. Consequently, DINCH may promote lipid accumulation and foam cell formation and may be linked to chronic inflammatory responses and vascular endothelial damage (Figure 9). In this study, we systematically integrated these key genes and pathways to construct a comprehensive AOP framework that provides a hypothetical basis for further experimental investigation of the molecular mechanisms by which DINCH exacerbates atherosclerosis. The AOP framework was constructed in a hypothesis-driven manner and provides a preliminary, inferential model to illustrate potential causal paths from molecular perturbations to atherosclerotic outcomes.

## 3. Discussion

Atherosclerosis is a global health challenge regulated by a complex interplay of genetic and environmental factors during its development [1]. In recent years, recognition of the health risks posed by traditional phthalate esters (PAEs) has grown, and the use of the alternative diisononyl cyclohexane-1,2-dicarboxylate (DINCH) has increased [16]. As a result, population exposure levels have risen, raising concern about long-term health effects [17]. Experimental studies showed that DINCH and its principal metabolite MINCH induced oxidative stress in macrophages and enhanced inflammatory responses at low concentrations; they promoted the release of proinflammatory cytokines such as TNF-α and IL-1β through activation of the NF-κB signaling pathway and potentially interfered with normal immune function [8]. Moreover, Eljezi confirmed that both primary and secondary metabolites of DINCH were significantly cytotoxic to L929 mouse cells [9]. Although DINCH was initially considered relatively safe, our computational toxicology analysis indicated hepatotoxicity, nephrotoxicity, and potential carcinogenicity. Its high lipophilicity (predicted Log P = 7.09) also indicated a strong potential for bioaccumulation and long-term health risks. The predicted carcinogenicity is a computational screening result with high uncertainty. Actual human exposure levels of DINCH are generally below the tolerable daily intake (TDI = 1 mg/kg body weight), so the real health risk remains low and needs further experimental verification. In this study, by integrating network toxicology, machine learning, and multidimensional bioinformatics strategies, we systematically revealed for the first time the potential molecular and immunological mechanisms by which DINCH exposure may contribute to the development of atherosclerosis.

Through a joint analysis of multiple databases, we identified 246 targets common to DINCH and atherosclerosis. GO and KEGG enrichment analyses showed that these targets were significantly enriched in immune–inflammatory processes, such as leukocyte migration and cytokine receptor binding, and in key metabolic and inflammatory pathways, including lipid and atherosclerosis, the PPAR signaling pathway, and the PI3K–Akt signaling pathway. These findings provide a systematic biological basis for the hypothesis that DINCH may promote atherosclerosis by disrupting lipid metabolic homeostasis and activating immune–inflammatory responses.

To identify core regulatory factors among many candidates, we applied two machine learning algorithms: LASSO regression and SVM-RFE [18]. This integrated strategy overcame the limitations of single-method approaches and yielded eight high-confidence core targets (CSF1R, CD36, CCL3, CCR2, ADAM8, TLR1, CTSS, MMP1). Validation in an independent dataset (GSE100927) confirmed that all eight genes were significantly upregulated in atherosclerotic plaque tissues. Further ROC curve analysis showed their excellent diagnostic performance (all AUC > 0.85), highlighting their potential as early biomarkers for the disease. Functionally, these targets form a cooperative pathogenic network: CSF1R regulates macrophage proliferation and survival [19]; CD36 mediates oxidized lipid uptake and foam cell formation [20]; and the CCL3–CCR2 axis drives monocyte and macrophage chemotaxis and recruitment [21,22]. ADAM8, TLR1, CTSS, and MMP1 contribute to inflammation activation, immune recognition and antigen presentation, and extracellular matrix remodeling, respectively. Together, this network indicates that DINCH may exacerbate atherosclerosis by promoting lipid accumulation, immune cell infiltration, and loss of vascular wall integrity.

Atherosclerosis is fundamentally a chronic inflammatory disease [23]. Our immune infiltration analysis (ssGSEA) showed that M0 macrophages, neutrophils, mast cells, and naïve CD4+ T cells were markedly increased in plaque tissues, indicating severe dysregulation of the local immune microenvironment. Importantly, the expression of the eight core targets correlated positively with infiltration by proinflammatory immune cells, including activated CD4+ memory T cells, γδ T cells, and M1 macrophages, and correlated negatively with infiltration by anti-inflammatory M2 macrophages. This proinflammatory–anti-inflammatory imbalance strongly suggests that DINCH may drive atherosclerosis progression by shaping a proinflammatory immune microenvironment. Considering that the ssGSEA method was primarily developed for tumor immune microenvironment studies, the immune infiltration results in the present study need to be interpreted prudently in the vascular microenvironment of atherosclerosis.

At the molecular interaction level, molecular docking showed that DINCH may bind to all eight core target proteins, with moderate but favorable binding affinity to CCR2 (binding free energy = −7.6 kcal/mol), suggesting a potential ability of DINCH to directly interfere with CCR2 function at the atomic level. This binding strength evaluation is based on computational docking results, and further experimental validation is required to confirm the actual interaction. A subsequent 100 ns molecular dynamics simulation confirmed the stability of the DINCH–CCR2 complex. Stable fluctuations in key physical indicators, including RMSD and Rg, provided kinetic evidence of stable binding [24]. Given CCR2’s central role in mediating monocyte and macrophage chemotaxis [25], we propose a positive feedback loop mechanism, DINCH, which acts as an initial stimulus, directly or indirectly activating macrophages and inducing secretion of proinflammatory factors such as IL-1β and TNF-α. These cytokines then activate the NF-κB signaling pathway [26] and upregulate CCR2 expression in macrophages via autocrine and paracrine signaling. Increased CCR2 expression enhances responsiveness to chemotactic signals, recruiting additional monocytes and macrophages and thereby amplifying and sustaining the inflammatory response. This positive inflammatory feedback loop initiated by DINCH and mediated jointly by NF-κB and CCR2 may play a potential role in its pro-atherosclerotic effect. KEGG enrichment analysis also indicates that the PPAR signaling pathway is a key pathway. As a core regulator of lipid metabolism, dysfunction of PPARα is a classic cause of atherosclerosis [27]. Some studies have shown that DINCH metabolites can activate PPARγ [28]. We therefore speculate that DINCH may accelerate disease progression by simultaneously disrupting PPARα function, which leads to lipid metabolism disorders, and PPARγ function, which may affect inflammatory responses and vascular function, thereby synergizing with the inflammatory pathway described above. In addition, enrichment of the PI3K–Akt signaling pathway suggests that DINCH may affect cell survival, proliferation, and metabolism, offering new insight into how it may exacerbate pathological changes in vascular wall cells.

We then constructed a comprehensive adverse outcome pathway (AOP) framework based on the above findings [29]. The framework systematically depicts the cascade that begins with DINCH exposure as the molecular initiating event; proceeds through perturbation of core targets, such as CCR2; disrupts key pathways, including lipid metabolism, PPAR signaling, and cytokine–cytokine receptor interaction as cellular/tissue-level key events; and ultimately results in atherosclerosis as the adverse outcome. This AOP provides an integrated theoretical model for understanding DINCH toxicity and indicates directions for subsequent experimental validation and risk assessment. As a hypothetical framework based on computational evidence, this AOP awaits future experimental validation.

From a public health perspective, the widespread use of DINCH in food packaging and medical devices raises concern because low-dose, long-term exposure may increase cardiovascular risk. The eight core targets identified in this study serve as potential molecular biomarkers for future population biomonitoring and early risk assessment. Moreover, the adverse outcome pathway (AOP) framework we constructed provides a theoretical model and scientific basis that regulatory agencies can use to evaluate the comprehensive health risks of alternative plasticizers. We call for a more comprehensive, mechanism-based, long-term safety reevaluation of these so-called “safe alternatives”.

The methodological strength of this study is the integration of multiple computational biology approaches, which creates a complete evidence chain from target prediction (network toxicology) and core-gene screening (machine learning) to interaction validation (molecular docking and molecular dynamics simulation). This multilevel integration strategy improves the reliability of the predictions. Nevertheless, the study’s conclusions must be interpreted with caution because all findings were derived from computational simulations and retrospective analyses of public databases. In future work, we will perform in vitro experiments, for example, treating human macrophages with DINCH and its metabolite MINCH to verify up-regulation of CCR2 expression and the release of downstream inflammatory factors. We will also carry out in vivo exposure experiments using animal models to confirm the pro-atherosclerotic effects of DINCH.

Based on current findings, future research should prioritize the following areas. First, experimentally validate core targets by performing gain- and loss-of-function studies in cell and animal models to determine the causal roles of CSF1R, CCR2, and related genes in DINCH toxicity. Second, address metabolite activity by including major DINCH metabolites (e.g., MINCH) in experimental designs to better represent in vivo exposure. Third, investigate epigenetic mechanisms to determine whether DINCH produces long-term regulation of these core genes via DNA methylation, histone modification, or other epigenetic pathways.

We recognize several limitations in this study. First, all findings are computational and require experimental validation; the conclusions should therefore be considered hypothesis-generating. Second, the ssGSEA method was originally developed for tumor studies, so its use in atherosclerotic tissues warrants some caution. Third, our molecular dynamics simulations did not include a membrane environment for CCR2, which may not fully reflect its native state. Fourth, the predicted binding energies from different scoring functions should be interpreted as relative rankings rather than absolute values. Finally, the proposed adverse outcome pathway is inferential and awaits experimental confirmation. Future studies incorporating metabolite exposure and in vivo models will be valuable to extend these findings.

## 4. Materials and Methods

### 4.1. Toxicity Analysis of DINCH

To comprehensively evaluate DINCH’s biological activity and potential toxicity, we applied a suite of computational chemistry methods. First, we retrieved the two-dimensional chemical structure (Canonical SMILES) for “DINCH” (diisononyl cyclohexane-1,2-dicarboxylate; BASF SE, Ludwigshafen, Rhineland-Palatinate, Germany) from the PubChem database (https://pubchem.ncbi.nlm.nih.gov/ accessed on 16 August 2025), which served as the primary input for subsequent analyses. We then employed two computational toxicology platforms to assess DINCH’s hazard profile: ProTox 3.0 (https://tox.charite.de/ accessed on 16 August 2025) to predict multiple toxicological endpoints, and ADMETlab 3.0 (https://admetlab3.scbdd.com/ accessed on 16 August 2025) to predict absorption, distribution, metabolism, excretion, and toxicity (ADMET) properties. By entering the Canonical SMILES of DINCH, these tools generated detailed predictions of toxicological parameters and pharmacokinetic properties, such as bioavailability and metabolic stability, thereby providing essential data for a comprehensive safety and health risk assessment of DINCH.

### 4.2. Collection of the Target of Diisononyl Cyclohexane-1,2-dicarboxylate

We retrieved the compound DINCH from the PubChem database to obtain its canonical SMILES, SDF structure file, and molecular weight. Based on the canonical SMILES, we restricted species to Homo sapiens in the Comparative Toxicogenomics Database (CTD, https://ctdbase.org/ accessed on 13 August 2025), SwissTargetPrediction (http://swisstargetprediction.ch/ accessed on 23 July 2025), and STITCH (http://stitch.embl.de/ accessed on 23 July 2025) to predict and collect potential targets. In STITCH, we applied an interaction score threshold of >0.7 to retain high-confidence results. This threshold (score > 0.7) is a widely accepted standard for identifying high-confidence compound–target interactions. For CTD and SwissTargetPrediction, only experimentally validated and top-ranked targets were retained to ensure reliability and reduce database bias (Table S5). We then took the union of predicted target lists from the different databases and mapped all entries to standardized gene symbols and UniProt KB identifiers via the UniProt database (https://www.uniprot.org/). After removing redundancies, we assembled the final set of potential DINCH targets.

### 4.3. Collection of Targets Related to Atherosclerosis

Using “atherosclerosis” as the keyword, we systematically retrieved potential target genes associated with atherosclerosis from the GeneCards (https://www.genecards.org/ accessed on 30 July 2025), OMIM (https://omim.org/ accessed on 30 July 2025), and TTD (https://db.idrblab.net/ttd/ accessed on 24 July 2025) databases. To enhance the relevance of targets to the disease, we applied thresholds based on each database’s recommended criteria or median scores (for example, the median “Relevance score” for GeneCards and the default scoring criteria for OMIM and TTD). We retained only genes with scores above the respective thresholds to construct an atherosclerosis-related target set. These thresholds were set according to database default standards and median scores to ensure disease relevance and minimize false positives. To identify potential common targets between DINCH and atherosclerosis, we intersected the DINCH predicted target set with the disease target set. We used the Venn diagram web tool (http://bioinformatics.psb.ugent.be/webtools/Venn/ accessed on 13 September 2025) to draw a Venn diagram and visualize the overlaps. The intersection targets were identified as the core candidate targets through which DINCH might induce atherosclerosis.

### 4.4. Functional Pathway Analysis of Target Genes

To elucidate the biological functions of the intersection targets between DINCH and atherosclerosis, we used the DAVID database (https://davidbioinformatics.nih.gov/ accessed on 8 September 2025) to perform Gene Ontology (GO) and Kyoto Encyclopedia of Genes and Genomes (KEGG) pathway enrichment analyses. The analysis included the three GO categories—biological processes, cellular components, and molecular functions—and KEGG signaling pathways, with Homo sapiens selected as the species background. GO terms and KEGG pathways showing significant enrichment were then selected using a threshold of FDR < 0.05.

### 4.5. Selection of Core Targets Based on Machine Learning Algorithms

We used two machine learning algorithms—the Least Absolute Shrinkage and Selection Operator (Lasso) regression [30] and Support Vector Machine–Recursive Feature Elimination (SVM-RFE) [31]—to identify core targets associated with DINCH-induced atherosclerosis. Lasso regression compresses and selects variables through L1 regularization to reduce overfitting, while SVM-RFE finds an optimal variable subset by iteratively eliminating features. We defined overlapping genes identified by both algorithms as the core targets.

The LASSO logistic regression model formula is:

$$\underset{\beta }{\min}\left\{-\frac{1}{n}\sum_{i=1}^{n}\left[y_{i}\beta^{T}x_{i}-\log\left(1+e^{\beta^{T}x_{i}}\right)\right]+\lambda {\left\|\beta \right\|}_{1}\right\}$$

For implementation, we ran Lasso regression with the “glmnet” R package using standardize = TRUE, alpha = 1, family = “binomial”, and nfolds = 10. 10-fold nested cross-validation was applied throughout feature selection and model training. To avoid information leakage, feature standardization and parameter tuning were performed independently within each training fold, without using test-fold data. All feature data were standardized prior to modeling. This dual-algorithm strategy improves the robustness and reliability of the identified core targets. For rigorous independent external validation, two completely independent atherosclerosis cohorts (GSE43292 and GSE28829)—which were not involved in any step of feature selection, model training, or parameter tuning—were utilized to assess the diagnostic performance of the gene signature. Receiver operating characteristic (ROC) curves and area under the curve (AUC) values were calculated to evaluate the sensitivity, specificity, and generalization ability of the signature across independent patient populations. Key parameters and the full machine learning R script are provided in the Supplementary Materials to ensure reproducibility.

### 4.6. Evaluation of Differential Expression of Core Genes and Diagnostic Accuracy

We downloaded the Gene Expression Omnibus (GEO) dataset GSE100927, which contains transcriptome data from atherosclerotic lesion tissues of the carotid artery, femoral artery, lower limb arteries, and lesion-free control tissues. Using this dataset, we compared mRNA expression profiles between the lesion and control groups. We applied thresholds of |log2 fold change| > 1 and adjusted *p*-value < 0.05 to identify significantly differentially expressed genes. To evaluate the diagnostic potential of the identified core targets, we constructed receiver operating characteristic (ROC) curves from the mRNA expression data of normal and atherosclerotic samples in GSE100927 and quantified classification accuracy by calculating the area under the curve (AUC).

### 4.7. Analysis of Immune Cell Infiltration

We used the GSVA software package (v1.46.0) to perform single-sample gene set enrichment analysis (ssGSEA) on the samples [32] to quantify immune cell infiltration. The LM22 gene signature, which defines 22 immune cell types, was used as the reference gene set. ssGSEA was selected instead of CIBERSORT because it calculates stable relative enrichment scores suitable for non-cancerous bulk atherosclerotic plaque tissue and performs reliably in inflammatory vascular tissues. To ensure robustness, we used the well-validated standard LM22 signature and followed established analytical protocols for vascular tissue. We employed the Mann–Whitney U test (Wilcoxon rank-sum test) for statistical analysis to evaluate the association between the core target genes identified by machine learning and immune cell infiltration levels [33]. All analyses and visualizations were carried out in the R environment (v4.2.0), and plots were generated with the ggplot2 package.

### 4.8. Molecular Docking

Based on the core targets identified previously, we retrieved the three-dimensional structures of the key proteins from the Protein Data Bank (PDB; http://www.rcsb.org/ accessed on 24 December 2025). All protein structures were checked for chain integrity, resolution, and missing loops. All protein structures were prepared for docking using standard protocols. Specifically, we removed all non-protein molecules (including water molecules, crystallographic ligands, and ions) from the PDB files. Missing amino acid residues and side chains were repaired using the “model” module in PyMOL 2.3.0 [34] or equivalent tools. Hydrogen atoms were added to the protein structures, and protonation states were assigned at physiological pH (7.4). Finally, energy minimization was performed to relieve steric clashes and ensure structural stability prior to docking. We obtained DINCH’s molecular structure from PubChem and optimized the small-molecule geometry with the MMFF94 force field in OpenBabel 3.1.1 [35] to obtain the lowest-energy conformation. The protonation states of both DINCH and target proteins were carefully examined and optimized prior to docking. Using AutoDock Tools 1.5.6 [36], we added hydrogen atoms to the proteins and to the ligand, defined the ligand’s rotatable bonds, and saved both as pdbqt files. All PDB IDs, chains, and preparation steps were fully verified. The binding site of CCR2 (PDB: 5T1A) was defined as the orthosteric ligand binding pocket in the crystal structure. The grid box was centered on the native ligand to cover the entire functional cavity. We configured docking parameters with the Grid module, selected semi-flexible docking, set exhaustiveness to 25, and chose the Lamarckian genetic algorithm. The docking protocol was verified by confirming DINCH bound to the classical functional pocket of CCR2, consistent with known ligand binding sites. No re-docking of reference antagonists or RMSD calculation was performed in this preliminary study; validation was based on correct occupancy of the known orthosteric pocket. Molecular docking was performed with AutoDock Vina 1.2.5 [37] to calculate binding free energies and generate result files. Finally, we visualized the lowest-energy complex conformation in PyMOL 2.3.0.

### 4.9. Molecular Dynamics Simulation

We used GROMACS 2022.3 [38,39] to perform molecular dynamics simulations of the DINCH–atherosclerosis receptor complex. The protein was described with the Amber14SB force field, and the ligand used the GAFF2 force field. The solvent was represented by the TIP4P water model, and a periodic water box with a 1.2 nm buffer was constructed. Long-range electrostatic interactions were computed with the particle-mesh Ewald (PME) method. Since this is a preliminary stability analysis, we used a water-only system without membrane embedding for efficient computational evaluation. Sodium and chloride ions were added by the Monte Carlo placement method to neutralize the system charge. Before the production simulations, the system energy was minimized and equilibrated in three steps. First, each system was minimized by the steepest-descent algorithm for 50,000 steps, stopping when the maximum force fell below 1000 kJ/mol/nm. Second, each system was pre-equilibrated for 50,000 steps with a 2 fs time step under constant particle number, volume, and temperature (310 K). Temperature was controlled using a V-rescale thermostat. Third, the entire system was pre-equilibrated for 50,000 steps with a 2 fs time step under constant particle number, pressure (1 bar), and temperature (310 K). Pressure was controlled using a Parrinello–Rahman barostat. After minimization and equilibration, a 100 ns molecular dynamics simulation was carried out without constraints using a 2 fs time step, and structural coordinates were saved every 10 ps.

### 4.10. Methodology for Constructing Adverse Outcome Pathways

Based on phenotypic and gene network analyses of DINCH and atherosclerosis, this study identified phenotypes that were significantly associated with both and thus considered them potential key events (KEs). Genes that were highly correlated with these phenotypes were regarded as possible molecular initiating events (MIEs). We then integrated hierarchical relationships among phenotypes and their biological contexts from the existing literature and, weighing the evidence and biological plausibility, gradually constructed an adverse outcome pathway (AOP) linking DINCH exposure to the development of atherosclerosis.

## 5. Conclusions

This study applied network toxicology and computational simulation to investigate potential targets and mechanisms by which DINCH influences atherosclerosis development. Initial screening identified 246 potential targets. Using machine learning, we then identified eight key genes—CSF1R, CD36, CCL3, CCR2, ADAM8, TLR1, CTSS, and MMP1—that may mediate DINCH-driven exacerbation of atherosclerosis. Molecular docking and molecular dynamics simulations showed that DINCH binds stably to these proteins, particularly CCR2. Based on these results, we constructed an adverse outcome pathway (AOP) framework to systematically describe how DINCH may promote atherosclerosis via effects on these targets. The findings suggest that the environmental pollutant DINCH may be an important risk factor for atherosclerosis progression and provide a theoretical basis for further research. However, these conclusions require validation through additional pharmacological and clinical experiments.

## Data Availability

The data presented in this study are available on request from the corresponding author. The data are not publicly available due to the need for further research utilization and subsequent project development. Gene expression datasets (GSE100927) can be accessed from the NCBI GEO repository (https://www.ncbi.nlm.nih.gov/geo/, accessed on 13 August 2025). The 3D structure of diisononyl cyclohexane-1,2-dicarboxylate (ligand) is available from the PubChem database (https://pubchem.ncbi.nlm.nih.gov/, accessed on 16 August 2025), and protein structures are available from the PDB database (https://www.rcsb.org/, accessed on 24 December 2025) or UniProt database (https://www.uniprot.org/, accessed on 24 December 2025). Data analysis was performed using R software 4.3.1. Custom code and processed data used in this study are available from the authors upon reasonable request.

## Supplementary Materials

The following supporting information can be downloaded at: https://www.mdpi.com/article/10.3390/ijms27114668/s1.
