# Supplementary material for: Exploring the Toxicological Relationship Between Diisononyl Cyclohexane-1,2-dicarboxylate and Atherosclerosis Through Network Toxicology, Machine Learning, and Multi-Dimensional Bioinformatics
Source: Int J Mol Sci. 2026 May 22;27(11):4668. doi: 10.3390/ijms27114668 (PMC13257353; doi:10.3390/ijms27114668)
Supplement: Supplementary file 1 [file ijms-27-04668-s001.zip › Supplementary Material.pdf]

Supplementary Material for

Exploring the Toxicological Relationship Between Diisononyl Cyclohexane-1,2-dicarboxylate and Atherosclerosis Through Network Toxicology, Machine Learning, and Multi-Dimensional Bioinformatics

Jingbo Cao [1,2,†], Ziyao Yang [1,2,†], Qi Zhang [1], Siwei Zou [3], Huning Zhang [1], Anning Yang [1,3,*] and Yue Sun [1,3,4,*]

1 General Hospital of Ningxia Medical University, School of Public Health, Ningxia Medical University, Yinchuan 750101, China; cao.jingbo@outlook.com (J.C.); yilin.2004@139.com (Z.Y.); 202304144040@nxmu.edu.cn (Q.Z.); 230240510143@nxmu.edu.cn (H.Z.)

2 The Second School of Clinical Medicine, Ningxia Medical University, Yinchuan 750101, China

3 NHC Key Laboratory of Metabolic Cardiovascular Diseases Research, Ningxia Medical University, Yinchuan 750101, China; 24132z210112@nxmu.edu.cn

4 Key Laboratory of Environmental Factors and Chronic Disease Control, Ningxia Medical University, Yinchuan 750101, China

* Correspondence: yanganning@nxmu.edu.cn (A.Y.); sunyue@nxmu.edu.cn (Y.S.)

† These authors contributed equally to this work.

Contents:

Table S1. Oral toxicity prediction results for DINCH.

Table S2. Toxicophore rules.

Table S3. ProTox -Prediction of toxicity of chemicals.

Table S4. Information on Receptor Proteins.

Figure S1. Binding Energies of Ligands and Receptors.

Table S5. Summary of databases used for DINCH target collection.

Table S1. Oral toxicity prediction results for DINCH

Name	DINCH
Moleweight	424.66
Number of hydrogen bond acceptors	4
Number of hydrogen bond donors	0
Number of atoms	30
Number of bonds	30
Number of rotable bonds	18
Molecular refractivity	127.55
Topological Polar Surface Area	52.6
octanol/water partition coefficient(logP)	7.09

Table S2. Toxicophore rules.

Property	Value	Decision
DILI	0.707	🔴
Skin Sensitization	0.965	🔴
Eye Irritation	0.721	🔴
Respiratory Toxicity	0.346	🟡
H-HT	0.068	🟢
AMES Toxicity	0.003	🟢
Rat Oral Acute Toxicity	0.261	🟢

The corresponding relationships of the three labels (green, yellow, red) are as follows: excellent, medium and poor.

Table S3. ProTox -Prediction of toxicity of chemicals.

**Classification**	**Target**	**Prediction**	**Probability**
Organ toxicity	Hepatotoxicity	Inactive	0.8
Organ toxicity	Neurotoxicity	Inactive	0.86
Organ toxicity	Nephrotoxicity	Active	0.54
Organ toxicity	Respiratory toxicity	Inactive	0.98
Organ toxicity	Cardiotoxicity	Inactive	0.64
Toxicity end points	Carcinogenicity	Active	0.77
Toxicity end points	Immunotoxicity	Inactive	0.99
Toxicity end points	Mutagenicity	Inactive	0.97
Toxicity end points	Cytotoxicity	Inactive	0.88
Toxicity end points	BBB-barrier	Active	0.87
Toxicity end points	Ecotoxicity	Inactive	0.58
Toxicity end points	Clinical toxicity	Inactive	0.73
Toxicity end points	Nutritional toxicity	Inactive	0.91
Molecular Initiating Events	Thyroid hormone receptor alpha (THRα)	Inactive	0.5
Molecular Initiating Events	Thyroid hormone receptor beta (THRβ)	Inactive	0.72
Molecular Initiating Events	Transtyretrin (TTR)	Inactive	0.57
Molecular Initiating Events	Ryanodine receptor (RYR)	Inactive	0.93
Molecular Initiating Events	GABA receptor (GABAR)	Inactive	0.64
Molecular Initiating Events	Glutamate N-methyl-D-aspartate receptor	Inactive	0.95

Molecular Initiating Events	Kainate receptor (KAR)	Inactive	1
Molecular Initiating Events	Acetylcholinesterase (AChE)	Inactive	0.8
Molecular Initiating Events	Constitutive androstane receptor (CAR)	Inactive	0.99
Molecular Initiating Events	Pregnane X receptor (PXR)	Inactive	0.55
Molecular Initiating Events	NADH-quinone oxidoreductase (NADHOX)	Active	0.5
Molecular Initiating Events	Voltage gated sodium channel (VGSC)	Inactive	0.9
Molecular Initiating Events	Na+/I- symporter (NIS)	Inactive	0.93

Table S4. Information on Receptor Proteins

Protein	PDB/Uniprot ID	Center (X,Y,Z)	Size (X×Y×Z)
ADAM8	4DD8	12, -7, 11	60×48×50
CCL3	3FPU	52, 85, 50	34×32×31
CCR2	5T1A	11, 23, 170	44×58×75
CD36	5LGD	-48, -35, 28	26×24×28
CSF1R	6T2W	19, 27, 2	65×62×63
CTSS	2H7J	12, 32, 15	45×59×55
MMP1	1SU3	8, -10, 40	20×20×20
TLR1	2Z7X	-31, 16, 26	75×83×92

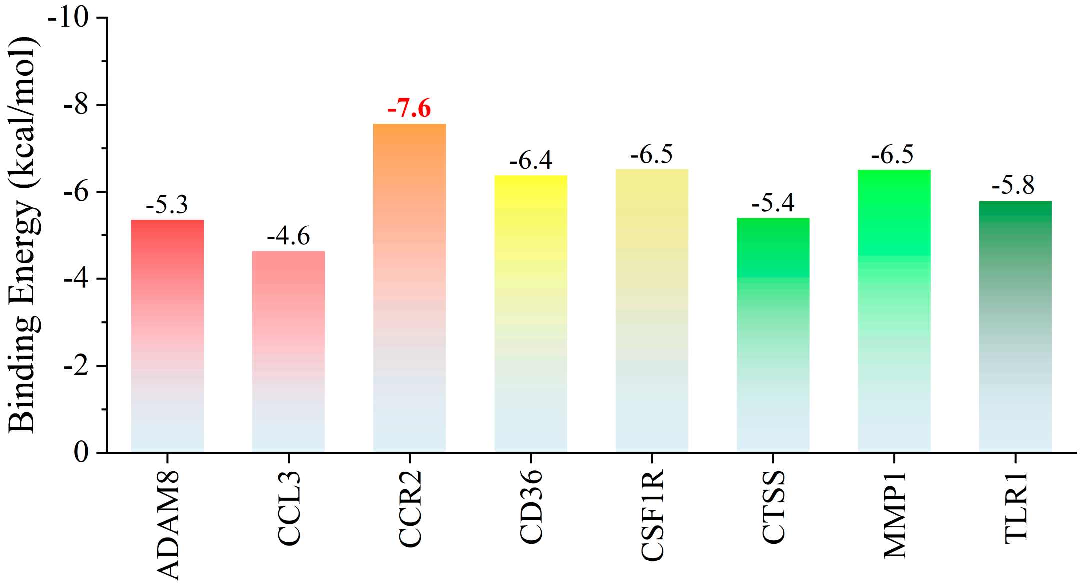

Figure S1. Binding Energies of Ligands and Receptors

Table S5. Summary of databases used for DINCH target collection.

**Database**	**Evidence Type**	**Species Processing**	**Confidence Level**
CTD	Manually curated (experimental evidence)	All species curated genes converted to human orthologs by STRING	High
SwissTargetPrediction	Structure-based target prediction	Homo sapiens	Medium
STITCH	High-confidence functional interaction (score > 0.7)	Homo sapiens	High
